# Social Determinant of Health Impact on Diabetes Device Use and Clinical Outcomes in Youth with Type 1 Diabetes

**DOI:** 10.1155/2023/4751595

**Published:** 2023-10-30

**Authors:** Emily R. Crain, Ryan Ramphul, Ashley M. Butler, Xiaofan Huang, Charles G. Minard, Maria J. Redondo, Daniel J. DeSalvo

**Affiliations:** ^1^Baylor College of Medicine, 1 Baylor Plaza, Houston, TX 77030, USA; ^2^UTHealth Houston School of Public Health, 1200 Pressler Street, Houston, TX 77030, USA

## Abstract

**Background:**

Youth with Type 1 diabetes (T1D) who are Black, Hispanic, or lower socioeconomic status (SES) have lower rates of diabetes device use, higher hemoglobin A1c (HbA1c), and higher rates of diabetic ketoacidosis (DKA). However, the associations of individual-level social determinants of health (SDoH) and neighborhood-level factors with device use and clinical outcomes are unknown. Area deprivation index (ADI) is a neighborhood level measure of SES reported in deciles (range 1–10 with 10 representing most deprived neighborhood).

**Methods:**

We evaluated the association of ADI and other SDoH factors with pump/continuous glucose monitor (CGM) use, HbA1c, and DKA in 1,461 youth with T1D (50% female, age 12.8 ± 3.6 years, HbA1c 8.7 ± 2.1%, 52% pump, 70% CGM) seen between October 1, 2020 and September 30, 2021 at a large pediatric diabetes center. Multiple logistic regression and multiple linear regression analyses were used to determine statistically significant associations adjusting for potential confounders.

**Results:**

Youth were less likely to use an insulin pump if they lived in a higher ADI neighborhood, were Black or Hispanic, had Medicaid or were uninsured, or received government assistance (e.g., Supplemental Security Income, Supplemental Nutritional Assistance Program). Youth were less likely to use a CGM if they lived in a higher ADI neighborhood, were Black or Hispanic, had Medicaid or were uninsured. Youth had higher risk of DKA event in the past year if they used government assistance, whereas pump and CGM use were associated with lower DKA risk. HbA1c (%) increased by 0.09 (95% CI: 0.05, 0.13) per unit increase in ADI. HbA1c was 0.62 lower (95% CI: −0.82, −0.42) in pump users vs. nonusers and 0.78 lower (95% CI: −0.99, −0.56) in CGM users vs. nonusers.

**Conclusions:**

Interventions that tailor care plans to address SDoH in families living in deprived neighborhoods may be needed to increase successful technology uptake, optimize HbA1c, and prevent DKA.

## 1. Introduction

The incidence of Type 1 diabetes (T1D) has been steadily increasing, with the steepest increases in African American, Hispanic, Asian American, and Pacific Islander racial/ethnic groups [1, 2]. Youth with lower annual household income, public health insurance, and who are Black or Hispanic are reported as having suboptimal diabetes outcomes including higher hemoglobin A1c (HbA1C) and more episodes of diabetic ketoacidosis (DKA) [3–9]. There are many complex reasons why these disparities exist including but not limited to structural racism, provider bias, patient preference, and barriers in access to quality healthcare in those of lower socioeconomic status (SES).

Use of diabetes technology including continuous glucose monitor (CGM) and insulin pump in diabetes management is associated with lower HbA1c compared to insulin injections and self-monitoring of blood glucose (SMBG) [6, 10–12]. The most recent guidelines from the International Society for Pediatric and Adolescent Diabetes (ISPAD) [13, 14] and the American Diabetes Association (ADA) [15] encourage insulin pump and CGM for self-management of diabetes in youth with T1D. However, individuals with lower SES and who are Black or Hispanic have lower rates of diabetes device utilization [3, 5, 6, 8, 9, 16–20].

Although racial/ethnic and socioeconomic disparities in diabetes device use and clinical outcomes have been well-documented, it remains unclear how to effectively address them through quality improvement initiatives, clinical research, and clinical care programs. Additionally, multiple family level social determinants of health including housing quality, food insecurity, reliable transportation, stable employment, and level of education, as well as neighborhood deprivation can affect health outcomes. Studies around the world have linked higher area deprivation in individuals with diabetes with higher HbA1c [21–23], higher rates of hospital admissions and DKA [24, 25], and higher rates of diabetes complications [26, 27]. However, the correlation between area deprivation and pediatric T1D outcomes and diabetes technology use has not been widely studied in the United States. Area deprivation index (ADI) is a geographically based measure of socioeconomic status in the United States that considers several measures, including income, employment, education, and housing quality in the neighborhood that one lives in the study of Kind and Buckingham [28]. ADI is reported in deciles from 1 to 10, where 1 represents the least deprived neighborhoods and 10 the most deprived. A previous study found that higher ADI was predictive of recurrent DKA admissions, with a more pronounced influence in pediatric than adult patients with T1D [29]. However, there is a paucity of data on how area deprivation and other family level social determinants of health impact insulin pump and CGM usage and other clinical outcomes in youth with T1D. Therefore, in this study we examined the association of race and ethnicity, insurance status, ADI score, and other family level social determinants of health with insulin pump and CGM use in youth with T1D at a large, academic, tertiary, urban hospital with a diverse patient population. Additionally, we examined the associations of insulin pump and CGM use with HbA1c levels and DKA rates while adjusting for demographic and social determinant of health variables [30].

## 2. Materials and Methods

The study was approved by the Baylor College of Medicine Institutional Review Board. We deployed the Epic® electronic medical record (EMR) population health management system for the Texas Children's Hospital (TCH) diabetes patient registry to generate a comprehensive data report. Inclusion criteria included T1D duration >1 year, age <19 years, having an address available to determine ADI score, and had at least one diabetes clinic visit with a social work assessment during the study period (October 1, 2020–September 30, 2021) to ensure that SDoH predictor variables were available. It is standard of care at our diabetes center for all patients with T1D to have a visit with a social worker on an annual basis. Recorded variables from the TCH diabetes patient registry included age, gender, race and ethnicity, duration of diabetes, insurance type, parents' preferred language, home address (which enabled the generation of ADI scores), insulin pump use, CGM use, most recent HbA1C (%) level, DKA episodes in the past year (both within and outside the TCH system), housing type, receipt of government assistance other than Medicaid (i.e., Supplemental Security Income (SSI), Special Supplemental Nutrition Program for Women Infants, and Children (WIC), Supplemental Nutrition Assistance Program (SNAP), public housing, or unemployment benefits), whether the family owned a motor vehicle, and history of child protective services (CPS) involvement with the family. Registry information on insulin pump and CGM use were generated from the last clinical encounter within the study period, wherein the provider routinely enters current device use into an EMR-based “Diabetes Flowsheet”.

### 2.1. Statistical Analysis

Demographics were summarized by mean with standard deviation, or frequency with percentages. Univariable logistic regression was used to identify patient characteristics (i.e., race and ethnicity, health insurance type, parents' preferred language, ADI score, housing type, receipt of government assistance other than Medicaid, whether the family owned a motor vehicle, and history of CPS involvement) that were significantly associated with insulin pump and CGM use. Multiple logistic regression was used to include all the significant factors from the univariable model, and backward selection by *p*-values was used to choose a reduced model. Simple linear regression was used to assess whether insulin pump use and CGM use were significantly associated with HbA1c level. Multiple linear regression was used to determine whether insulin pump use and CGM use were still significantly associated with HbA1c after adjusting for other patient characteristics. Logistic regression was used to assess if insulin pump and CGM use were significantly associated with having a DKA episode in the past year. Multiple logistic regression was used to assess whether insulin pump use and CGM use were still significant after adjusting for other patient characteristics. A significance level of *p*-value < 0.05 was used. All analyses were conducted using RStudio Team (2020). RStudio: Integrated Development for R. RStudio, PBC.

## 3. Results

Among 1,881 youth with established T1D seen at TCH in the study period, 1,461 had been seen by a social worker and had an address available to determine the ADI score. This study cohort of 1,461 T1D youth was 50.0% female with mean age of 12.8 ± 3.6 years, duration of diabetes of 5.6 ± 3.5 years, HbA1C of 8.7 ± 2.1%, and 113 (7.7%) had an episode of DKA in the past year. The mean ADI score for the study cohort was 4.1 ± 2.5 including 711 (49%) with in the least deprived range (ADI score of 1–3), 578 (40%) in the moderately deprived range (ADI score of 4–7), and 172 (12%) in the most deprived range (ADI score of 8–10). Overall, 765 (52.4%) youth were using an insulin pump and 1,030 (70.5%) were using a CGM for diabetes management. The demographic characteristics are summarized in Table 1.

### 3.1. Insulin Pump and CGM Use

**Figure 1:**
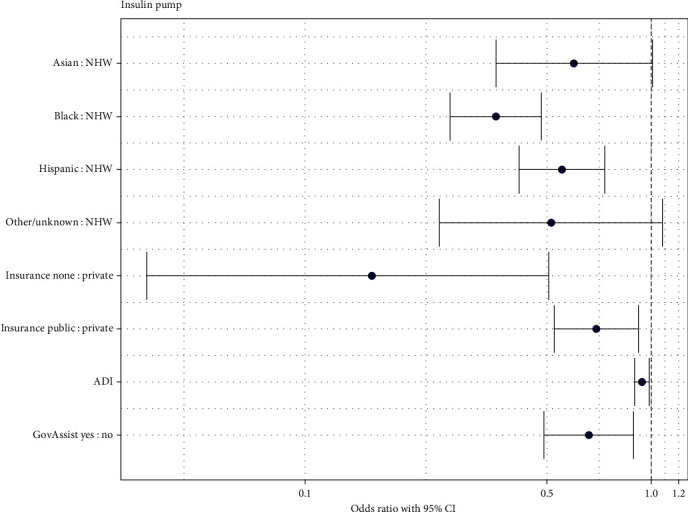
Odds ratios of insulin pump use.

In univariable logistic regression, all variables of interest (race and ethnicity, insurance type, preferred language, ADI score, housing type, use of government assistance other than Medicaid, mode of transportation, and history of CPS involvement) were significantly associated with insulin pump use. In the reduced model assessing patient characteristics associated with pump use, race and ethnicity (*p* < 0.001), insurance status (*p*=0.001), ADI score (*p*=0.014), and receipt of government assistance other than Medicaid (*p*=0.006) were significantly associated with using an insulin pump. The odds ratios of insulin pump use according to these predictor variables are displayed in Figure 1. Compared to non-Hispanic White youth, non-Hispanic Black (OR: 0.36, 95% CI: 0.26, 0.48), and Hispanic (OR: 0.55, 95% CI: 0.42, 0.73) youth were less likely to use an insulin pump after adjusting for insurance status, ADI score, and receipt of government assistance. Compared to the youth with private insurance, youth with public insurance (OR: 0.69, 95% CI: 0.53, 0.92), and uninsured youth (OR: 0.16, 95% CI: 0.04, 0.51) were less likely to use an insulin pump after adjusting for race and ethnicity, ADI score, and receipt of government assistance other than Medicaid. Youth who used government assistance other than Medicaid (OR: 0.66, 95% CI: 0.49, 0.89) were less likely to use an insulin pump after adjusting for race and ethnicity, insurance status, and ADI score. For every 1-unit increase in ADI score, youth were 0.94 times as likely to use insulin pump (95% CI: 0.89, 0.99) after adjusting for race and ethnicity, insurance status, and receipt of government assistance other than Medicaid.

**Figure 2:**
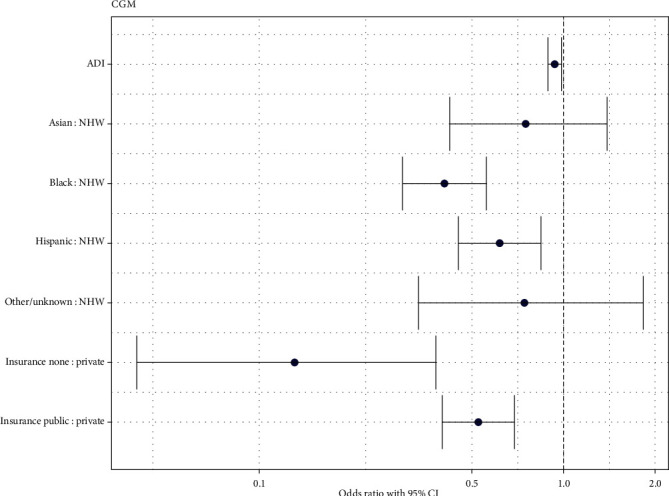
Odds ratios of CGM use.

In univariable logistic regression, all variables of interest (race and ethnicity, insurance type, preferred language, ADI score, housing type, use of government assistance other than Medicaid, mode of transportation, and history of CPS involvement) were significantly associated with CGM use. In the reduced model assessing patient characteristics associated with CGM use, ADI score (*p*=0.011), race and ethnicity (*p* < 0.001), and insurance status (*p* < 0.001) were significantly associated with using CGM. The odds ratios of CGM use according to these predictor variables are displayed in Figure 2. For every 1-unit increase in ADI score, youth were 0.94 times as likely to use CGM (95% CI: 0.89, 0.99) after adjusting for race and ethnicity and insurance status. Compared with non-Hispanic White youth, non-Hispanic Black (OR: 0.41, 95% CI: 0.29, 0.56), and Hispanic (OR: 0.62, 95% CI: 0.45, 0.84) youth were less likely to use a CGM after adjusting for insurance status and ADI score. Compared to the youth with private insurance, youth with public insurance (OR: 0.53, 95% CI: 0.39, 0.69), and uninsured youth (OR 0.13, 95% CI: 0.04, 0.38) were less likely to use a CGM after adjusting for race and ethnicity and ADI score.

### 3.2. DKA Risk

**Figure 3:**
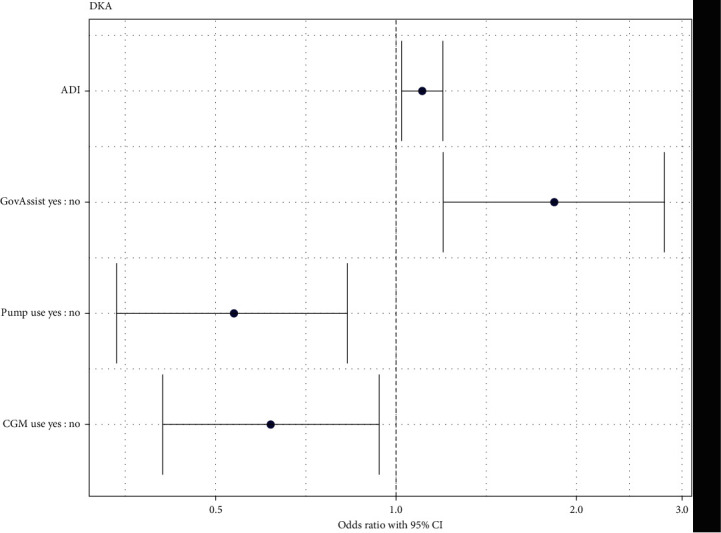
Odd ratios of having a DKA episode in the past year.

In univariable logistic regression, all variables other than preferred language (*p*=0.278) and transportation (*p*=0.066) were significantly associated with having a DKA episode in the last year. In the reduced model assessing patient characteristics associated with DKA, ADI score (*p*=0.013), government assistance other than Medicaid (*p*=0.005), insulin pump use (*p*=0.006), and CGM use (*p*=0.023) were significantly associated with having a DKA episode in the past year. The odds ratios of having a DKA episode in the past year according to significant predictor variables are displayed in Figure 3. For every 1-unit increase in ADI score, youth were 1.11 times as likely to use have had a DKA episode in the past year (95% CI: 1.02, 1.19) after adjusting for receipt of government assistance other than Medicaid, insulin pump use, and CGM use. Youth who used government assistance other than Medicaid were more likely (OR: 1.84, 95% CI: 1.19, 2.81) to have had a DKA episode in the past year after adjusting for ADI score, insulin pump use, and CGM use. Youth using an insulin pump were less likely (OR: 0.54, 95% CI: 0.34, 0.83) to have had a DKA episode in the past year after adjusting for ADI score, receipt of government assistance other than Medicaid, and CGM use. Youth using a CGM were less likely (OR: 0.62, 95% CI: 0.41, 0.94) to have had a DKA episode in the past year after adjusting for ADI score, receipt of government assistance other than Medicaid, and insulin pump use.

### 3.3. Glycemic Control (HbA1c %)

**Figure 4:**
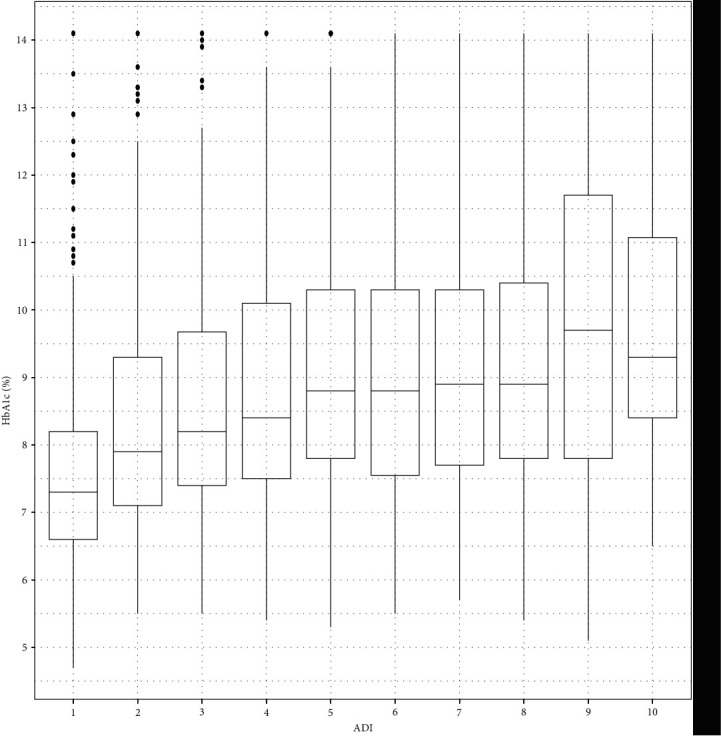
Box plot of HbA1c (%) for each ADI decile. legend: the horizontal bars in the boxes represent the median HbA1c for each ADI decile, the lower and upper boundaries of each box represent the 25th and 75th HbA1c percentiles, and the dots represent outliers, which were calculated by Q3 + 1.5 ^*∗*^ (Q3–Q1) for upper fence and Q1–1.5 ^*∗*^ (Q3–Q1) for lower fence.

Compared to non-Hispanic White youth, HbA1c among non-Hispanic Black youth was 1.2 higher (95% CI: 0.94, 1.46, *p* < 0.001) after adjusting for insurance status, ADI score, use of government assistance other than Medicaid, insulin pump use, and CGM use. HbA1c was 0.11 higher in Hispanic youth compared to the non-Hispanic White youth, but this was not statistically significant (95% CI: −0.13, 0.35) after adjusting for insurance status, ADI score, use of government assistance other than Medicaid, insulin pump use, and CGM use. Compared to youth with private insurance, HbA1c among youth with public insurance was 0.38 higher (95% CI: 0.14, 0.62) after adjusting for race and ethnicity, ADI score, use of government assistance other than Medicaid, insulin pump use, and CGM use. For every 1-unit increase in ADI score, HbA1c increased by 0.09 (95% CI: 0.05, 0.13) after adjusting for race and ethnicity, insurance status, use of government assistance other than Medicaid, insulin pump use, and CGM use (Figure 4). HbA1c of youth using government assistance other than Medicaid was 0.48 higher (95% CI: 0.23, 0.73) than youth not using government assistance other than Medicaid after adjusting for race and ethnicity, insurance status, ADI score, insulin pump use, and CGM use. HbA1C of youth using an insulin pump was 0.62 lower (95% CI: −0.82, −0.42) than patients not using an insulin pump, and the HbA1c of youth using a CGM was 0.78 lower (95% CI: −0.99, −0.56) than youth not using a CGM after adjusting for race and ethnicity, insurance status, ADI score, and use of government assistance other than Medicaid.

## 4. Discussion

In this cross-sectional analysis of pediatric patients with T1D at a large, diverse diabetes center, differences in diabetes device use existed across race and ethnicity and health insurance, but there were also notable differences based on the neighborhood in which one lives. Youth living in a neighborhood with a higher ADI score indicating neighborhood deprivation in income, employment, education, and housing quality were less likely to use insulin pump and/or CGM even after adjusting for sociodemographic factors such as race and ethnicity and insurance status. For every 1-unit increase in ADI score, youth were about 6% less likely to use insulin pump or CGM even after adjusting for race and ethnicity and insurance status. Differences in access to diabetes devices across race and ethnicity and insurance status in youth with T1D have been well-documented [8, 9, 18–20, 31, 32], but this is the first study indicating that neighborhood-level deprivation may also play a key role.

Disparities in insulin pump and CGM use were found between Hispanic and non-Hispanic Black patients compared to the non-Hispanic White youth. This is consistent with the previous studies showing higher diabetes device use in non-Hispanic White patients compared with the Black and Hispanic youth [8, 9, 18–20, 31, 32]. Black youth were also found to have higher HbA1c compared to the non-Hispanic White youth, which is also consistent with the previous data [20]. It has been suggested that racial/ethnic disparities in diabetes technology use may be perpetuated by unconscious bias by providers assessing Black and Hispanic youths' readiness for diabetes devices [32].

Interestingly, there was no significant association between race and ethnicity and DKA risk when adjusting for other variables, which is a contrast from the other studies [7, 9]. This may be in part because our study adjusted for multiple family level and geographic SDOH variables, which may have more of an impact on DKA risk than race and ethnicity. In our cohort, the only variables associated with increased risk of DKA were ADI and receipt of government assistance other than Medicaid (i.e., SSI, WIC, SNAP, public housing, or unemployment benefits), suggesting that those most disproportionately impacted by SDoH are at highest risk for DKA. DKA is a potentially avoidable complication of T1D that carries a significant risk for morbidity and mortality with a high-economic burden on families, health care systems, and payers [33]. The findings from our study suggest that SDoH assessment with a focus on neighborhood deprivation and receipt of government assistance other than Medicaid could be clinically useful for identifying patients with high risk of DKA so that interventions can be tailored to these families to help avoid this costly and potentially fatal event.

Disparities in insulin pump and CGM use were also found in patients who had public insurance compared to the patients with private insurance. This is consistent with the previous studies showing higher diabetes technology use in youth with private insurance and higher annual household income [8, 19]. Texas Medicaid provides comprehensive coverage for insulin pump therapy and CGM devices, so coverage cannot explain these disparities in our state. This suggests there may be unmeasured variables contributing to these disparities such as patient preference or provider bias in assessing pump or CGM readiness.

A unique aspect of this study was the examination of the association of family level social determinants of health with diabetes technology use and diabetes outcomes. These family level social determinants of health included housing type, receipt of government assistance such as SSI, WIC, SNAP, public housing, or unemployment benefits, whether the family owned a motor vehicle, and history of CPS involvement. Many of these specific SDoH factors and their relationship to diabetes technology have not been previously reported. In our study, most of these family level SDoH factors were not significantly associated with diabetes device use, glycemic control as measured by HbA1c, or the likelihood of DKA event in the past year when adjusting for race and ethnicity, insurance status, and ADI score. However, use of government assistance including SSI, WIC, SNAP, public housing, or unemployment benefits was associated with lower odds of insulin pump use and higher odds of DKA episode in the past year. Use of such government assistance indicates some type of family stress such as food insecurity, housing instability, unemployment, or a disabled family member. This stress may preclude patients/families or providers from taking on what they perceive as an additional burden with new diabetes technology, or the diabetes care team may not address diabetes devices during clinic encounters because they are addressing what they perceive as more pressing psychosocial and clinical concerns. For families of youth with T1D with higher social needs, a more informed understanding of their wider living context may help inform tailored care plans and interventions to optimize technology uptake, improve glycemic control, and prevent DKA.

Another unique aspect of this study was the use of ADI score to evaluate disparities in diabetes technology use and clinical outcomes. A recent study showed that higher ADI score was associated with higher odds of DKA readmission in both adult and pediatric patients [29]. This is consistent with our study which found the odds of DKA admission in the past year increased with higher ADI score. For every 1-unit increase in ADI score, youth were about 10% more likely to have had a DKA event in the past year, indicating that neighborhood deprivation in income, employment, education, and housing quality may contribute to DKA risk. Similarly, living in a more deprived neighborhood was associated with having poor glycemic control with about 0.1% higher HbA1c for every 1-unit increase in ADI score.

This is the first study specifically assessing association of ADI score with diabetes technology use and glycemic control in youth with T1D. A recent study in adults with T1D showed lower odds of CGM discussion with their healthcare provider in CGM-naïve adults who lived in neighborhoods with the highest ADI scores [17]. This is consistent with the findings in our study of lower odds of CGM use with higher ADI score. These findings of lower rates of technology use, higher risk of DKA, and suboptimal glycemic control with higher ADI score suggest that there are important neighborhood influences impacting clinical outcomes in pediatric diabetes. This highlights the important role of the comprehensive diabetes care team including social workers to holistically support patients and families. Novel Interventions in Children's Healthcare (NICH) is a community-based program that assigns an interventionist to a family of a child with a chronic medical condition such as T1D and a high degree of social risk. The interventionist helps connect the family with resources for basic needs as well as providing ongoing social and emotional support and behavioral interventions to improve adherence to their treatment regimen [34]. Community-based programs such as NICH have the potential to significantly improve diabetes outcomes in youth.

In our study, insulin pump and CGM use were both associated with lower HbA1C and lower odds of having a DKA event in the past year, even after adjusting for race and ethnicity, insurance status, and ADI score. This is consistent with previous studies reporting improved diabetes outcomes with use of technology [6, 31, 35]. Unfortunately, however, our data suggests that many barriers to use of diabetes devices exist. Studies have shown the existence of implicit bias among providers in recommending diabetes technology less often to patients in non-White racial and ethnic groups and those with public insurance [36, 37]. Further research is needed in diverse youth with T1D in areas of high ADI to obtain real-world and qualitative data to analyze the impact of provider bias, patient preference, and/or other factors in initiating and sustaining diabetes technology use.

This study has notable limitations. The cross-sectional study design does not allow determination of causality between social determinants of health and diabetes technology use or between diabetes technology use and diabetes outcomes. Only patients with established T1D who had a social work visit in the 1-year period of our study were included (1,461 out of 1,881 total T1D patients), which may have excluded some patients who had barriers to attending clinic appointments and may be at risk for suboptimal diabetes outcomes. Additionally, our data only captured device use at the time of the last clinical encounter in the study period and did not capture how consistently insulin pump and/or CGM were being used. Additional limitations are that the study did not include parental income or level of education, which are other important factors in diabetes technology use [9, 32, 38].

These findings show multiple variables impacting diabetes device use and clinical outcomes in youth with T1D including race and ethnicity, insurance type, and neighborhood-level factors. Addressing these barriers through comprehensive clinical and community-based programs including school nurse partnerships, community health workers, and peer support may promote diabetes technology use and could have a significant impact on improving diabetes outcomes. Diabetes care teams should consider the impact of neighborhood-level deprivation in income, employment, education, and housing quality when caring for youth with T1D.

## Figures and Tables

**Table 1:** Demographic characteristics for the 1,461 youth with T1D with ADI score and SDoH screen in the 1-year study period.

Characteristic	*N* (%)
*Age* <6 years 6 to <13 years 13 to <19 years	60 (4%) 424 (29%) 977 (67%)
*Gender* Female Male	730 (50%) 731 (50%)
*Race and ethnicity* Non-Hispanic White Non-Hispanic Black Hispanic Asian Other/unknown	661 (45%) 228 (20%) 401 (27%) 69 (5%) 31 (2%)
*Insurance type* Private Public None	803 (55%) 643 (44%) 15 (1%)
*Primary language of parent(s)* English Spanish Other	1,306 (89%) 141 (10%) 14 (1%)

## Data Availability

Data can be made available upon request by emailing the corresponding author at desalvo@bcm.edu.
